# Psychosocial determinants of active living: the role of social capital, public space use, and barriers to sport in Mexico City

**DOI:** 10.3389/fspor.2026.1912419

**Published:** 2026-09-23

**Authors:** Rafael Peñaloza Gómez, María del Pilar Méndez Sánchez, Eduardo David Núñez Hernández, Jose Carlos Jaenes Sanchez, Jesús Maya Mendoza, Andres Caloso Ruiz, Sonora Reyes de la Garza

**Affiliations:** 1 Facultad de Estudios Superiores Zaragoza, Universidad Nacional Autónoma de México, Mexico City, Mexico; 2 Universidad Pablo de Olavide, Seville, Spain; 3 Escuela Superior de Educación Física, Mexico City, Mexico; 4 Centro Deportivo Israelita, Mexico City, Mexico; 5 Universidad Autonoma de Nuevo Leon Facultad de Organizacion Deportiva, San Nicolás de los Garza, Mexico

**Keywords:** active living, barriers to sport, Mexico City, physical activity participation, public space use, social capital, subjective well-being, urban safety

## Abstract

**Introduction:**

Active living in urban environments depends not only on individual motivation or infrastructure, but also on the social conditions that shape the everyday use of public spaces. This study analyzed the relationships among social capital, urban safety, public space use, barriers to sport, subjective well–being, and physical activity participation in an urban Mexican sample.

**Methods:**

A quantitative, cross–sectional study was conducted with 837 adults who completed an online self–report questionnaire. Descriptive analyses, Pearson correlations, structural equation modeling, and binary logistic regression models were performed.

**Results:**

Social capital was positively associated with public space use and showed the strongest association with subjective well–being. Urban safety was not directly associated with public space use when examined together with social capital. Model fit was mixed, and the structural model explained little variance in barriers to sport (*R*² = .019). Barriers to sport showed a small negative association with subjective well–being and were associated with lower odds of physical activity participation. Public space use was associated with higher odds of physical activity participation and, among physically active participants, with engaging in physical activity in public rather than private spaces.

**Discussion:**

In this sample, social cohesion, trust, regional identity, and public space use were relevant correlates of active living and subjective well–being.

## Introduction

The World Health Organization (WHO) (1) defines physical activity as any bodily movement produced by skeletal muscles that requires energy expenditure. Such movements occur while travelling from one place to another, working, performing household tasks, and engaging in leisure-time activities.

From this perspective, physical activity can be understood not only as structured exercise but also as an everyday practice embedded within urban environments. Open public spaces constitute important settings for examining these practices, as they include parks and green areas as well as squares, promenades, and other publicly accessible open spaces (2). Consequently, urban environments may either promote or hinder physical activity depending on characteristics such as proximity, size, accessibility, and quality (3, 4).

In this context, public space use is closely related to the ways in which individuals incorporate these settings into their daily lives. Space appropriation may be understood as the process through which individuals transform a physical environment into a meaningful place through intentional use, familiarity, permanence, care, and identification with specific locations (5). It is also associated with the ways in which people interact with their surroundings, make use of them, and develop both functional and symbolic bonds with particular places (6, 7). From this perspective, regular physical activity in parks, squares, streets, promenades, and other open spaces may foster place appropriation by encouraging repeated presence, environmental familiarity, and social interaction.

Public space use depends not only on the physical characteristics of a setting but also on how these characteristics are perceived by users, particularly with regard to safety. When individuals perceive a space as safe, they are more likely to use it frequently, spend more time there, and participate in recreational and community-based activities, thereby fostering processes of place appropriation, place attachment, and social cohesion. Conversely, perceptions of insecurity may discourage the use of public spaces and reduce opportunities for social interaction and physical activity within them (8, 9).

Perceived safety refers to individuals' judgments about safety, risk, and potential threats. These judgments are influenced by conditions such as crime, urban infrastructure, green spaces, transportation, and traffic (10, 11). Here, perceived safety referred to whether participants viewed nearby settings as safe, risky, or threatening. These settings included neighborhoods, parks, squares, residential surroundings, streets, and other public spaces (12, 13).

Perceived safety has also been associated with physical activity participation and public space use. Castillo-Paredes et al. (14) and Pérez-Tejera et al. (13) found that individuals tend to use parks, recreational areas, and open spaces more frequently when they perceive them as safe, whereas perceptions of insecurity may discourage activities such as walking, running, or active transportation within urban environments. Furthermore, several indicators of perceived safety, including crime, public space safety, and traffic conditions, have been associated with higher levels of active mobility. Therefore, existing evidence suggests that perceived safety is an important factor for understanding participation in physical activity within urban environments (15).

In the context of physical activity, structural and contextual barriers are particularly important, as they include conditions such as accessibility, the availability of suitable spaces, perceived insecurity, costs, travel time, and the quality of the urban environment. Perceived barriers to physical activity refer to individuals' evaluations of potential obstacles to engaging in health-promoting behaviors (16). These barriers may be classified as follows (17): (1) intrapersonal barriers, such as activity preferences, stress, perceived competence, previous experiences, and motivation; (2) interpersonal barriers, such as the influence of exercise partners, friends, or family members, as well as body image concerns; and (3) structural barriers, such as lack of time, financial resources, or accessibility.

Studies conducted in Mexico have reported that adults perceive several barriers to physical activity participation, including lack of time (18, 19), lack of motivation, preference for sedentary activities, dislike of physical activity, health-related issues, lack of safe and adequate spaces, and the perception that physical activity is not a family priority (18). Other reported barriers include limited support from family and friends, lack of knowledge, and lack of encouragement (19). Therefore, barriers should not be understood solely as a matter of individual willpower; rather, they also reflect urban, family, social, and environmental inequalities that shape participation in physical activity and, consequently, access to experiences related to health, social interaction, and well-being.

Diener and Ryan (20) defined subjective well-being as the subjective evaluations individuals make about the quality of their own lives. They further argued that these evaluations may be positive or negative and may involve judgments and feelings related to life satisfaction, interest, engagement, life events, work, relationships, health, leisure, and other relevant life domains. Moreover, subjective well-being serves as an important criterion for assessing psychological development and mental health (21).

Likewise, physical activity has been associated with higher levels of subjective well-being. When performed in public spaces and urban green areas, its benefits may extend to social and psychological domains by promoting social interaction, contact with nature, and community participation, thereby contributing to a more positive evaluation of one's life (22, 23).

Public space use therefore involves more than physical access and perceived safety; it is also related to the social relationships and meanings attached to urban settings. Social capital is relevant to this relationship because it encompasses networks, norms, and trust that facilitate coordination and cooperation for mutual benefit (24). Within public spaces, these resources may support interpersonal interaction, community participation, and shared use. Gao et al. (25) identified social cohesion, trust, participation, reciprocity, neighborhood satisfaction, and social networks as dimensions of social capital commonly associated with physical activity.

These dimensions refer to distinct aspects of community life. Social cohesion concerns positive relationships, belonging, and orientation toward the common good (26). Trust and reciprocity involve expectations of reliability and mutual exchange (27, 28), while participation and social networks concern relationships and involvement beyond the family (29–31). Neighborhood satisfaction, by contrast, evaluates the residential environment (32). Although distinct, these dimensions help characterize the social context in which public spaces are used and evaluated.

Regional identity adds a place-based component to this social context. It refers to shared characteristics, values, and narratives associated with a region and its inhabitants (33). Identification with a community or territory can strengthen belonging and the meanings attached to everyday places. Regional identity therefore connects the relational components of social capital with place appropriation, because both concern how people relate to places and to others who inhabit them (34, 35).

The study examined public space use for physical activity within a set of related individual, social, and environmental conditions rather than as an isolated behavior. Social capital was treated as a contextual construct that included relational resources (trust and cohesion), regional identity, satisfaction with public space, and awareness of urban improvements. These indicators represent related, but not interchangeable, features of the social and shared urban environment. The analysis addressed the relationships among social capital, perceived urban safety, public space use, barriers to sport participation, and subjective well-being, as well as their associations with physical activity participation and activity in public spaces.

## Methods

### Study design

This was a quantitative, cross-sectional correlational study. The analytical model, based on the literature reviewed above, included social capital, perceived urban safety, public space use, perceived barriers to physical activity and sport participation, physical activity participation, and subjective well-being. A structural equation model was estimated first; logistic regression models were then used to examine the likelihood of participating in physical activity and of engaging in such activity in public spaces.

### Participants

The sample consisted of 837 participants aged 18–74 years (M = 27.3, SD = 10.6), with a median age of 22 years. Participants were primarily residents of urban areas in Mexico. A non-probability convenience sampling strategy was used

Of the total sample, 58.2% (*n* = 487) identified as women, 40.6% (*n* = 340) identified as men, and 10 participants preferred not to answer. Regarding educational attainment, most participants reported undergraduate studies (68.8%), followed by high school education (23.5%). Smaller proportions reported postgraduate studies (*n* = 32), secondary education (*n* = 28), and primary education (*n* = 4).

Most participants (79.9%) reported having no children. Regarding perceived social class, 306 participants identified themselves as belonging to a high social class (36.6%), 520 to a middle social class (62.1%), and 11 to a low social class (1.3%). Regarding physical activity and sport participation, 590 participants reported engaging in some form of physical activity or sport (70.5%), whereas 247 participants reported not doing so (29.5%). The main reason for engaging in physical activity, reported by 510 participants, was physical and mental health. Other reasons included social interaction and companionship, physical appearance, self-esteem, enjoyment, and the development of physical skills.

### Procedure

Data were collected using a self-report questionnaire administered online through Google Forms. Prior to participation, individuals were provided with an informed consent form describing the general purpose of the study, the voluntary nature of participation, the right to withdraw at any time, the confidential and anonymous handling of the information, and the exclusive academic and scientific use of the data. Participants were also informed that participation involved completing a questionnaire that took approximately 30 min.

By continuing with the questionnaire, participants confirmed that they had read and understood the information provided and agreed to participate voluntarily.

### Ethics statement

This study involving human participants was reviewed by the Ethics Committee of the Facultad de Estudios Superiores Zaragoza, Universidad Nacional Autónoma de México (UNAM). All procedures were conducted in accordance with the ethical principles for research involving human participants. Participants were informed about the general purpose of the study, the voluntary nature of their participation, their right to withdraw at any time, and the confidential and anonymous handling of their responses. Electronic informed consent was obtained before participants accessed the questionnaire. No personally identifiable information was collected, and the data were used exclusively for academic and scientific purposes.

### Measures

The self-report questionnaire consisted of sociodemographic sections, questions on physical activity and sport participation, and scales designed to assess psychosocial and urban variables related to active living, public space use, and subjective well-being.

The first section collected sociodemographic information, including age, educational attainment, marital status, sex, number of children, occupation, perceived social class, city, and borough or municipality. Age was treated as a continuous variable. Educational attainment was recorded across five ordered levels: primary school, secondary school, high school, undergraduate degree, and postgraduate degree. Marital status included the following categories: single, married, cohabiting, divorced, and widowed. Sex included the options male, female, and prefer not to answer. Perceived social class was recorded as high, middle, or low.

Physical activity participation was recorded as a dichotomous variable. Weekly frequency was organized into practice ranges; length of participation included options ranging from less than one year to lifetime participation; and place of practice included public, private, school, community, household, and other settings. Reasons for engaging in physical activity included physical and mental health, self-esteem, physical appearance, social interaction and companionship, enjoyment, and the development of physical skills. Cronbach's alpha and McDonald's omega coefficients were calculated to assess the internal consistency of the scales used.

Perceived urban safety, awareness of urban improvement initiatives, and trust in social and institutional actors were assessed using items adapted from the 2024 National Survey on Victimization and Public Safety Perception (ENVIPE 2024), developed by the Instituto Nacional de Estadística y Geografía (36).

Perceived urban safety was assessed using 12 items rated on a five-point response scale. The items asked participants about their perceptions of safety in different everyday settings, such as the home, workplace, streets, and other urban spaces. For the analyses, four items specifically referring to safety in public spaces were separated to create a specific indicator of perceived public space safety. The remaining items were used to represent overall perceived urban safety. Internal consistency for the full perceived urban safety scale was adequate, with a Cronbach's alpha of.908 and a McDonald's omega of.910.

Awareness of actions to improve public spaces was assessed using 11 items referring to actions carried out during the previous year, such as the construction or maintenance of parks and sports courts, improvements in street lighting, initiatives to improve family income, and actions addressing unemployment, among others. Responses were recorded on a five-point scale. Internal consistency indicators for this study were good (*α* = .918; *Ω* = .921).

Trust in social and institutional actors was assessed using items referring to the degree of trust in neighbors, coworkers, business or school associates, family members, friends, and other actors. Responses were recorded on a five-point scale ranging from complete distrust (1) to complete trust (5). Internal consistency was adequate (*α* = .920; *Ω* = .931).

Satisfaction with public space was assessed using a seven-item measure developed for this study. Item content was selected by a subject-matter specialist on the research team, drawing on the categories of urban settings used in ENVIPE 2024. Participants rated their satisfaction with parks and gardens, public transportation, sidewalks, streets and avenues, public squares, community centers, and forests or ecological reserves. Responses were recorded on a five-point Likert-type scale ranging from 1 = very dissatisfied to 5 = very satisfied. The overall score was the mean of the seven items, with higher values indicating greater satisfaction. Cronbach's alpha and McDonald's omega indicated acceptable internal consistency in the present sample (*α* = .875; *Ω* = .879).

Public space use was assessed with a four-item measure developed through the same content-selection procedure. The instructions asked participants to consider visits for physical activity, walks with family or friends, picnics, and social interaction. The items recorded how often they visited parks or gardens, ecological reserves or forests, public squares, and community centers. Response categories were 0 = never, 1 = once a year, 2 = once a month, 3 = three or more times a month, 4 = once or twice a week, and 5 = four or more times a week. The overall score was the mean of the four items, with higher values indicating more frequent public space use. Internal consistency was acceptable in the present sample (*α* = .777; *Ω* = .786). Neither measure was pilot-tested or examined in a separate construct-validation study; both were analyzed as observed mean composite scores rather than as separate latent measurement models.

Regional identity was assessed using the Revised Regional Identity Scale (RIS-2R) developed by Asún, Zúñiga, and Morales (37). This 17-item instrument was designed to assess sense of regional belonging using a five-point Likert-type response scale ranging from strongly disagree (1) to strongly agree (5). For this study, items were combined into a global regional identity score, with higher values indicating stronger regional identity. Internal consistency was adequate (*α* = .952; *Ω* = .956).

Social cohesion was assessed using the Neighborhood Cohesion Instrument developed by González-Tapia and Reyes-Lagunes (38). This scale measures components associated with sense of community, neighborhood attraction, and neighborhood relationships through 14 items. Responses are recorded on a five-point Likert-type scale ranging from 1 = strongly disagree to 5 = strongly agree, with higher values indicating greater neighborhood social cohesion. Cronbach's alpha and McDonald's omega values were adequate (*α* = .890; *Ω* = .904).

Barriers to sport, including aspects related to fatigue or laziness, body image, and social physique anxiety, among others, were assessed using the Self-Report of Barriers to Physical Exercise Practice (ABPEF) developed by Niñerola, Capdevila, and Pintanel (39). The instrument consists of 17 items rated on a scale from 0 to 10, where 0 indicates low probability and 10 indicates high probability. Higher scores indicate greater perceived barriers to physical exercise participation. The scale showed adequate internal consistency (*α* = .944; *Ω* = .945).

Subjective well-being was assessed using the Short Version of the Subjective Well-Being Scale (EBS-8) developed by Calleja and Mason (40). The scale evaluates two components: life satisfaction (*α* = .940; *Ω* = .940) and positive affect (*α* = .922; *Ω* = .924). Each component consists of four items rated on a seven-point Likert-type scale. For life satisfaction, response options ranged from 1 = strongly disagree to 7 = strongly agree; for positive affect, response options ranged from 1 = rarely to 7 = always.

### Data analysis

Statistical analyses were conducted using R version 4.5.2 and RStudio Build 218. Descriptive statistics were calculated for all study variables. Frequencies and percentages were obtained for categorical variables, whereas means, standard deviations, medians, minimum values, and maximum values were calculated for continuous variables. Pearson correlations were also estimated. Univariate distributions were examined using skewness and kurtosis; absolute values were below 1.0 for all study variables. Missingness among the variables reported in Table 1 ranged from zero to two cases. The structural equation model used full-information maximum likelihood to retain all available observations, whereas the logistic regression models used complete cases for the variables included in each model.

**Table 1:** Descriptive statistics of the variables used in the study.

Variable	n	M	SD	Median	Min.	Max.	Skewness	Kurtosis
Satisfaction with public space	837	2.60	0.84	2.57	1	5	0.15	−0.28
Public space use	837	1.74	0.99	1.50	0	5	0.60	0.12
Overall city safety	836	2.60	0.92	3.00	1	5	0.07	−0.12
Public space safety	837	3.05	0.74	3.08	1	5	−0.11	−0.10
Actions to improve public spaces	837	2.13	0.83	2.00	1	5	0.83	0.53
Trust in the social environment	836	2.50	0.74	2.46	1	5	0.51	0.06
Regional identity	837	2.88	0.98	2.88	1	5	0.09	−0.67
Social cohesion	835	2.86	0.80	2.86	1	5	0.18	−0.08
Barriers to sport	836	3.19	2.37	2.71	0	10	0.73	−0.21
Life satisfaction	837	3.74	0.96	4.00	1	5	−0.54	−0.09
Positive affect	837	3.76	0.92	4.00	1	5	−0.53	−0.03

n, number of participants; M, mean; SD, standard deviation; Min., minimum value; Max., maximum value.

Subsequently, a structural equation model was specified using the lavaan package. The model included three latent factors: social capital, urban safety, and subjective well-being. Social capital was represented by satisfaction with public space, trust in the social environment, regional identity, social cohesion, and awareness of urban improvement initiatives. Urban safety was represented by overall city safety and safety in public spaces. Subjective well-being was represented by life satisfaction and positive affect.

The model was estimated using robust maximum likelihood. Overall fit was evaluated using the chi-square statistic, comparative fit index, Tucker–Lewis index, root mean square error of approximation, and standardized root mean square residual. Two binary logistic regression models were also estimated. The first examined the likelihood of participating in physical activity or sport; the second, restricted to participants who reported physical activity, examined the likelihood of practicing in public rather than private spaces. Factor scores for social capital and urban safety were obtained from the structural model and entered in both logistic regressions together with public space use and barriers to sport. Adjusted versions of the two models also included age, sex, educational attainment, and perceived social class. Age was entered as a continuous variable; sex, educational attainment, and social class were entered as categorical variables, with women, undergraduate education, and middle social class as the respective reference categories. Participants who selected ‘prefer not to answer' for sex were excluded from the adjusted models.

Logistic regression diagnostics included convergence, evidence of complete separation, multicollinearity, linearity in the logit, calibration, and influential observations. Multicollinearity was evaluated using variance inflation factors; linearity for continuous predictors was examined with Box–Tidwell terms and comparisons with natural cubic spline specifications. Calibration was evaluated with Hosmer–Lemeshow tests, and influence with Cook's distance. Because no *a priori* sample-size calculation was available in the study records, sample adequacy was evaluated retrospectively. For the SEM (36 df), an RMSEA-based sensitivity calculation following MacCallum, Browne, and Sugawara (41) indicated that 273 observations would provide 80% power at *α* = .05 to distinguish close fit (RMSEA = .05) from poor fit (RMSEA = .08); the available sample was 837. The SEM estimated 41 free parameters, yielding 20.4 cases per parameter. For the adjusted logistic regressions, the smaller outcome categories contained 241 and 276 observations for 12 slope parameters, corresponding to conservative ratios of 20.1 and 23.0 outcome observations per parameter, respectively (42).

## Results

### Descriptive statistics and correlations

The sample included 837 participants aged 18–74 years (M = 27.3, SD = 10.6; media*n* = 22). Women represented 58.2% of the sample (*n* = 487), men 40.6% (*n* = 340), and 10 participants preferred not to answer. Most participants reported undergraduate education (68.8%), and 70.5% (*n* = 590) reported engaging in physical activity or sport.

Descriptive statistics for the main study variables are presented in Table 1. Mean scores were 2.60 (SD = 0.84) for satisfaction with public space, 1.74 (SD = 0.99) for public space use, 2.60 (SD = 0.92) for overall city safety, and 3.05 (SD = 0.74) for public space safety. Barriers to sport had a mean of 3.19 (SD = 2.37) on the 0-to-10 scale. Mean scores were 3.74 (SD = 0.96) for life satisfaction and 3.76 (SD = 0.92) for positive affect.

Pearson correlations are presented in Table 2. Satisfaction with public space was correlated with public space use (r = .386), overall city safety (r = .335), public space safety (r = .502), actions to improve public spaces (r = .513), trust in the social environment (r = .433), regional identity (r = .349), and social cohesion (r = .363); all *p* < .01.

**Table 2:** Pearson correlations among the main study variables.

Variable	1	2	3	4	5	6	7	8	9	10	11
1. Satisfaction with public space	—										
2. Public space use	.386**	—									
3. Overall city safety	.335**	.138**	—								
4. Public space safety	.502**	.329**	.423**	—							
5. Actions to improve public spaces	.513**	.413**	.333**	.501**	—						
6. Trust in the social environment	.433**	.284**	.343**	.537**	.536**	—					
7. Regional identity	.349**	.281**	.193**	.300**	.352**	.380**	—				
8. Social cohesion	.363**	.274**	.201**	.366**	.369**	.446**	.565**	—			
9. Barriers to sport	.102**	.078*	-.003	.028	.049	.039	.065	.065	—		
10. Life satisfaction	.169**	.221**	.027	.224**	.193**	.265**	.344**	.368**	-.083*	—	
11. Positive affect	.152**	.200**	.046	.239**	.203**	.277**	.343**	.380**	-.125**	.809**	—

* *p* <.05; **p <.01.

Public space use was correlated with overall city safety (r = .138), public space safety (r = .329), actions to improve public spaces (r = .413), trust in the social environment (r = .284), regional identity (r = .281), social cohesion (r = .274), life satisfaction (r = .221), and positive affect (r = .200); all *p* < .01.

Life satisfaction and positive affect were strongly correlated (r = .809, *p* < .01). Both were correlated with trust in the social environment (r = .265 and.277), regional identity (r = .344 and.343), and social cohesion (r = .368 and.380), respectively; all *p* < .01. Barriers to sport were negatively correlated with life satisfaction (r = −.083, *p* < .05) and positive affect (r = −.125, *p* < .01).

### Structural equation model

A structural equation model was then estimated to evaluate the relationships among social capital, urban safety, public space use, barriers to sport, and subjective well-being. The model included three latent variables: social capital, urban safety, and subjective well-being.

Model fit was mixed: *χ*^2^(36) = 273.89, *p* < .001; robust CFI = .917; robust TLI = .873; robust RMSEA = .090, 90% CI [.080,.100]; SRMR = .054. The CFI and SRMR were within commonly used ranges, whereas the TLI and RMSEA indicated limitations in overall fit. Standardized factor loadings were significant for all indicators. Loadings ranged from.569 to.711 for social capital, from.519 to.814 for urban safety, and from.882 to.917 for subjective well-being. Social capital was positively associated with public space use (*β* = .633, *p* < .001), whereas urban safety did not show a significant direct effect on this variable (*β* = −.154, *p* = .244). The model explained 25.8% of the variance in public space use.

Explained variance was low for barriers to sport (R^2^ = .019). Social capital showed the strongest positive association with subjective well-being (*β* = .740, *p* < .001), while barriers to sport showed a small negative association (*β* = −.080). The association with urban safety was also negative (*β* = −.345, *p* = .058). The model explained 24.6% of the variance in subjective well-being. Standardized estimates for the full model appear in Figure 1.

**Figure 1:**
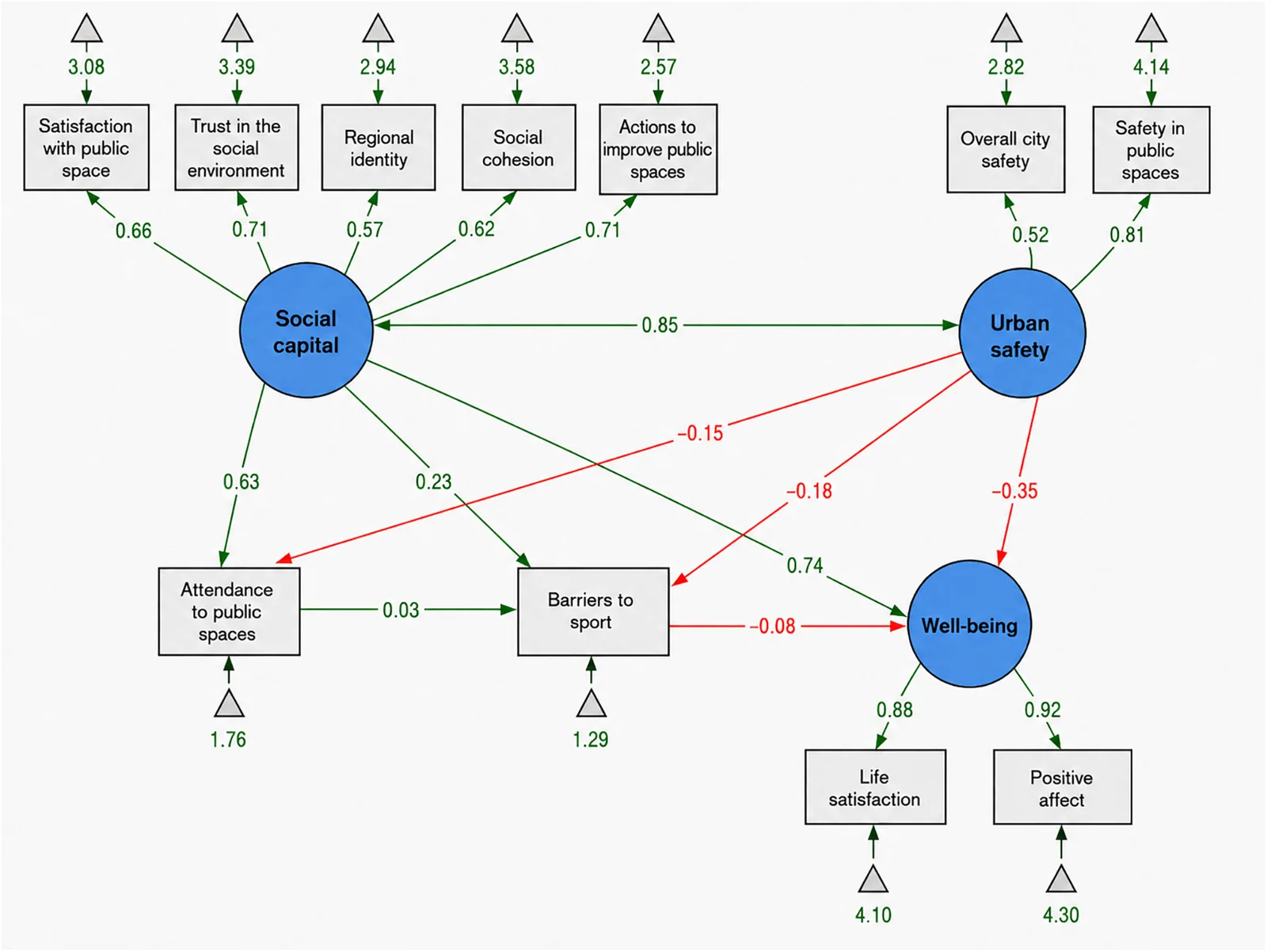
Structural equation model of the relationships among social capital, urban safety, public space use, barriers to sport, and subjective well-being.

### Physical activity participation and practice in public spaces

Binary logistic regression models were used to examine associations between the psychosocial variables and physical activity or sport participation. In the first model, public space use was associated with higher odds of engaging in physical activity or sport (OR = 1.32, *p* = .007), whereas barriers to sport were associated with lower odds of participation (OR = 0.80, *p* < .001). Social capital and urban safety were not significantly associated with participation in this model. In the sociodemographically adjusted model (*n* = 826), public space use remained positively associated with physical activity participation [adjusted odds ratio [aOR] = 1.25, 95% CI [1.01, 1.53], *p* = .039], and barriers to sport remained negatively associated with participation [aOR = 0.79, 95% CI (0.74, 0.85), *p* < .001]. Social capital [aOR = 2.55, 95% CI (0.92, 7.07), *p* = .071] and urban safety [aOR = 0.49, 95% CI (0.16, 1.54), *p* = .224] remained nonsignificant. Among the control variables, men had higher odds of participation than women [aOR = 1.64, 95% CI (1.17, 2.29), *p* = .004], whereas participants with secondary education had lower odds than those with undergraduate education [aOR = 0.36, 95% CI (0.15, 0.83), *p* = .016]. Age, the remaining educational contrasts, and perceived social class were not significantly associated with participation.

In the second model, restricted to participants who reported engaging in physical activity or sport, public space use was associated with higher odds of engaging in physical activity in public rather than private spaces (OR = 1.38, *p* = .003). Social capital, urban safety, and barriers to sport were not significantly associated with the practice setting. In the corresponding adjusted model (*n* = 556), public space use remained associated with higher odds of engaging in physical activity in public rather than private spaces [aOR = 1.46, 95% CI (1.17, 1.83), *p* < .001]. Social capital [aOR = 1.39, 95% CI (0.46, 4.22), *p* = .559], urban safety [aOR = 0.73, 95% CI (0.20, 2.59), *p* = .622], and barriers to sport [aOR = 1.07, 95% CI (0.98, 1.16), *p* = .118] were not significantly associated with the practice setting. Older age [aOR = 1.023 per year, 95% CI (1.004, 1.043), *p* = .020] and being a man rather than a woman [aOR = 2.04, 95% CI (1.43, 2.92), *p* < .001] were associated with higher odds of practicing in public rather than private spaces; educational attainment and perceived social class were not significantly associated with the practice setting.

Both adjusted logistic models converged and yielded finite coefficients and standard errors, with no indication of complete separation. Hosmer–Lemeshow tests did not indicate lack of calibration [*χ*^2^(8) = 14.26, *p* = .075; *χ*^2^(8) = 7.42, *p* = .493], and no case had a Cook's distance greater than 1. Multicollinearity was concentrated in the social capital and urban safety factor scores, which were strongly correlated (r = .932 and.936 across the two analytic samples) and had variance inflation factors ranging from 8.41 to 10.54; values for all remaining terms were 1.49 or lower. Models entering these two scores separately did not alter the positive association of public space use or render either factor score significant. Linearity diagnostics were satisfactory except for a modest departure for barriers in the participation model (spline likelihood-ratio *p* = .045; Box–Tidwell *p* = .034). A spline sensitivity specification retained a monotonic negative association and left the public space use estimate essentially unchanged (aOR = 1.25, *p* = .038). The linear barriers odds ratio is therefore retained as a summary across the observed range and should be interpreted cautiously.

Across the two regressions, public space use was associated with physical activity participation and, among active participants, with practicing in public rather than private settings. Perceived barriers were associated with lower odds of participation, while the structural model accounted for little of their variance.

## Discussion

The study examined relationships among social capital, urban safety, public space use, barriers to sport, and subjective well-being, as well as their associations with physical activity participation and activity in public spaces. Social capital was strongly associated with public space use and subjective well-being, while urban safety was not directly associated with public space use. Barriers to sport were negatively associated with subjective well-being and the odds of physical activity participation. The TLI and RMSEA nevertheless indicate that these patterns should be interpreted cautiously.

The positive relationship between social capital and public space use is consistent with Putnam (24, 28), who argued that social networks, trust, and norms of reciprocity facilitate cooperation and participation in collective activities. Gao et al. (25) likewise noted that dimensions of social capital, including social cohesion, trust, and community participation, are often associated with more active lifestyles. In this analysis, social capital comprised satisfaction with public space, trust in the social environment, social cohesion, regional identity, and awareness of actions to improve public spaces. These indicators formed the social capital construct and were positively associated with everyday public space use.

Regarding space appropriation, several authors have suggested that appropriation involves the construction of functional and symbolic bonds with places through use, familiarity, permanence, and social interaction (5–7). Higher levels of cohesion, trust, and community identification may facilitate the incorporation of public spaces into everyday practices. Public spaces can then become socially meaningful places for encounters, interaction, and shared experiences, in addition to serving as settings for physical activity.

Regional identity also showed positive associations with public space use, subjective well-being, and other indicators of social capital. Previous research similarly suggests that a sense of belonging to a community or territory may strengthen bonds with everyday places and foster community participation (33–35).

Contrary to expectations, urban safety did not show a significant direct association with public space use when it was examined jointly with social capital. At the bivariate level, however, both overall city safety (r = .138, *p* < .01) and public space safety (r = .329, *p* < .01) were positively correlated with public space use. Because urban safety and social capital were entered simultaneously, the nonsignificant direct path represents the association unique to safety after accounting for social capital. This does not mean that safety was unrelated to public space use. Previous studies have likewise documented more frequent use of parks, squares, and other open spaces when they are perceived as safe (13–15). Although modeled separately, the two variables were closely related. Social capital may partly incorporate perceptions of safety, and this overlap reduced the precision of their separate coefficients.

In Mexico City, perceived safety is linked to victimization and fear of crime. ENVIPE 2024 documents concern about insecurity and changes in daily activities due to fear of crime (43). These experiences vary across unequal urban conditions, including disparities in neighborhood access to green space and micro-scale segregation that affects the use and appropriation of public space (44, 45). A citywide model may obscure differences among areas, although victimization and segregation were not measured here.

Social capital also showed a strong association with subjective well-being. Research has linked social relationships, belonging, and community participation to quality of life and psychological well-being (20, 21). The correlations further showed that regional identity, social cohesion, and trust in the social environment were positively associated with life satisfaction and positive affect. In this sample, subjective well-being covaried with both individual and social characteristics.

Barriers to sport were negatively associated with subjective well-being and the likelihood of physical activity participation. The structural model explained only 1.9% of their variance, indicating that the social and urban predictors in the model accounted for little of the differences in perceived barriers. The barrier measure includes proximal personal concerns such as fatigue or laziness, body image, and social physique anxiety, whereas the predictors emphasized community and urban conditions. Other influences not represented in the model, including time demands, motivation, health, costs, and social support, may be more closely related to these barriers (17–19).

Public space use was related both to physical activity participation and, among active participants, to practicing in public spaces. Previous research has likewise identified public spaces as accessible settings for physical activity and active mobility (2–4). The association with community social resources, however, indicates that access alone does not account for how participants used these settings.

Public space use cannot be understood only through the physical characteristics of the urban environment. Infrastructure, accessibility, and safety remain relevant, but social ties, trust, community cohesion, and belonging were also related to how participants used and valued public spaces. The study does not establish intervention effects; it identifies material and community conditions that should be examined together.

## Conclusions

In Mexico City, public space use and physical activity were associated with both urban conditions and community resources. Local action should address the conditions that make neighborhood public spaces usable, not only their physical availability.

Local governments and urban planners should consider maintenance, accessibility, perceived safety, and community participation as related concerns. Public health programs could connect physical activity promotion with equitable opportunities to use neighborhood public spaces. The cross-sectional design and Mexico City's socio-spatial heterogeneity mean that these findings can inform local assessment but do not demonstrate intervention effects.

### Limitations

One limitation of this study is that some scales were analyzed using global composite scores, even though the original instruments include specific dimensions. This decision was made to maintain a parsimonious model focused on the general relationships among psychosocial and urban variables. Future research could examine the dimensional structure of these instruments and evaluate alternative models that distinguish the relative contribution of specific dimensions of regional identity, social cohesion, social trust, and perceptions of the urban environment.

The convenience sample consisted mainly of young adults and participants with university-level education, which limits the generalizability of the findings to other population groups. The results should not be assumed to represent older adults, people with lower educational attainment, or residents from socioeconomic groups that were sparsely represented in the sample. Because the questionnaire was administered online, participation may also have favored people with regular internet access and greater familiarity with online surveys. The self-report measures may also be subject to memory, perception, or social desirability biases.

The study did not include objective indicators of the urban environment or direct measures of physical activity. Physical activity was recorded through self-reported participation and practice setting rather than a standardized measure of frequency, intensity, and duration. The findings therefore concern participation and setting, not an objectively quantified activity level. Future research could integrate geospatial information, built environment audits, or objective physical activity records to strengthen the study of factors associated with public space use and active lifestyles.

## Data Availability

The raw data supporting the conclusions of this article will be made available by the authors, without undue reservation.
